# Compromised Dynamic Cerebral Autoregulation in Patients With Idiopathic Rapid Eye Movement Behavior Disorder: A Case-Control Study Using Transcranial Doppler

**DOI:** 10.3389/fpsyt.2020.00051

**Published:** 2020-02-19

**Authors:** Shan Lv, Zan Wang, Xin Sun, Hang Jin, Jia Liu, Fang Deng, Yudan Lv, Meiyan Jia, Zhen-Ni Guo, Yi Yang

**Affiliations:** ^1^Department of Neurology, The First Hospital of Jilin University, Changchun, China; ^2^Shenzhen Institutes of Advanced Technology, Chinese Academy of Sciences, Shenzhen, China; ^3^Clinical Trial and Research Center for Stroke, Department of Neurology, The First Hospital of Jilin University, Changchun, China

**Keywords:** idiopathic rapid eye movement sleep behavior disorder, cerebral autoregulation, cerebral hemodynamics, transcranial Doppler, transfer function

## Abstract

**Background:**

Patients with idiopathic rapid eye movement behavior disorder (IRBD) have been suggested to exhibit altered cerebral perfusion and abnormal cerebral blood flow, which imply a possibility of cerebral autoregulation (CA) impairment. We aimed to investigate the dynamic CA (dCA) in patients with IRBD during wakefulness and to explore the correlations between dCA parameters and clinical measurements.

**Methods:**

We assessed the dCA capability of 30 patients with IRBD and 36 sex- and age-matched healthy controls by using transcranial Doppler and finger plethysmography. CA function was evaluated by transfer function analysis based on spontaneous oscillation of cerebral blood flow and arterial blood pressure. Transfer function parameters (phase difference and gain) were used to quantify the CA.

**Results:**

No significant differences were observed between the right and left middle cerebral artery dCA parameters (phase difference and gain) of both groups. Patients with IRBD had significantly lower phase difference than the healthy controls, indicating their impaired CA capability. Besides, the value of gain in patients with IRBD was higher than the healthy controls, but the difference did not reach statistical level.

**Conclusions:**

CA function is compromised in patients with IRBD during wakefulness, which might be an intermediate link between IRBD and neurological symptoms.

## Introduction

Idiopathic rapid eye movement (REM) sleep behavior disorder (IRBD) is a parasomnia characterized by loss of normal skeletal muscle atonia and dream-enacting behaviors during REM sleep, which affect 0.5% to 2% of the general populations and 5% to 13% of adults older than 60 years (1). RBD is not only frequent in patients with neurodegenerative diseases, but also considered to be a prodromal sign, especially of those with alpha-synuclein deposition such as Parkinson's disease (PD) and Lewy body dementia (LBD). It has been demonstrated that patients with IRBD have abnormal brain perfusion (2–4), which is associated with neurological symptoms such as cognitive impairment (5), as well as markers of neurodegeneration (4). This abnormality probably implies the disorder to maintain the mechanism for cerebral blood flow (CBF). However, the exact mechanisms are still unknown and worthy of further discussion.

Adequate cerebral perfusion, maintained by precise regulation of CBF, is essential for normal brain function (6). Cerebral autoregulation (CA) is a physiological mechanism of the brain to maintain sufficient CBF despite changes in blood pressure (BP)/cerebral perfusion pressure. Impairment of CA leads to alteration in cerebral perfusion accompanied by oscillation of BP, which has a role in both cerebrovascular diseases and several neurodegenerative diseases such as PD (7) and Alzheimer's disease (8, 9). To the best of our knowledge, CA in patients with IRBD has not yet been discussed.

In the current study, we hypothesized that CA is impaired in the patients with IRBD, which might be an intermediate link between IRBD and neurological symptoms related to cerebral perfusion alteration. To test this hypothesis, we compared the dynamic CA (dCA) ability using transcranial Doppler and finger plethysmography between the patients with untreated IRBD and volunteers without IRBD and analyzed the correlation between dCA parameters and clinical measurements.

## Methods

The study design was approved by the ethics committee of the First Hospital of Jilin University under the guidelines of the Declaration of Helsinki, and an informed consent form was signed by all the participants.

### Subjects

Patients diagnosed with IRBD by a sleep specialist from the Department of Neurology, First Hospital of Jilin University were recruited consecutively in this study. The diagnostic criteria of IRBD were according to the International Classification of Sleep Disorders, 3rd Edition: (1) repeated episodes of behavior or vocalization that are either documented by polysomnography (PSG) to arise from REM or are presumed to arise from REM based on reports of dream enactment, and (2) evidence of REM sleep without atonia (RSWA) on PSG (as defined in the scoring manual) (10). Exclusion criteria included the presence of neurodegenerative diseases such as PD, multiple system atrophy (MSA), LBD; a Mini-Mental State Examination score below 26; obstructive sleep apnea syndrome (defined as apnea-hypopnea index >10/h) (11, 12); history of cerebrovascular diseases or cardiovascular disease; taking antidepressant medication; insufficient bilateral temporal bone windows for insonation of the middle cerebral artery (MCA). None of the patients had been treated previously for the disorder. Thirty-six age-matched healthy volunteers without neurological diseases or sleep complains were recruited as controls. Participants of the control group were all tested negative by RBD Single-Question Screen (13). The exclusion criteria for the control group was the same as that for the IRBD patients. All the participants underwent a detailed neurological examination to exclude the presence of neurodegenerative disorders.

### Video PSG

All patients underwent overnight video PSG recording (Compumedics E Series EEG/PSG Recording System, Australia) in the sleep laboratory to confirm the diagnosis of IRBD. PSG monitoring included standard electroencephalography (C3-A2, O2-A1), bilateral electrooculography, chin muscle electromyography, and electrocardiography. A body position sensor was also attached. Respiration was monitored using a nasal cannula and a thoracic strain gauge. Blood oxygen saturation was continuously recorded by a transcutaneous finger pulse oximeter. Sleep stages 1–3 and REM sleep were scored on the basis of the American Academy of Sleep Medicine Scoring Manual Updates for 2017 (AASM, Version 2.4) (14, 15).

### RSWA Scoring

The occurrence of the first REM epoch was used to determine the onset of a REM sleep period. The termination of REM sleep periods was identified by the occurrence of an EEG feature indicative of another stage (K complex, sleep spindle, or EEG sign of arousal) or by the absence of REMs during six consecutive 30-sec epochs. RSWA was scored according to the AASM manual (version 2.4) (15). Tonic activity was scored when EMG activity was present at least twice the amplitude of the baseline EMG (measured during non-REM sleep) or >10 μV during more than 50% of total 30-sec epoch. Phasic activity was defined as all muscle activity during 0.1–5 s that exceeded four times the background EMG activity in >50% of 3-sec miniepochs within a 30-sec epoch. Tonic RSWA and phasic RSWA percentage were calculated as the percentage of REM epochs that show tonic or phasic RSWA.

### Autonomic Tests

Autonomic tests were performed using supine-to-standing TCD test described previously (16). Briefly, participants were told to maintain in a supine position for 3 min, then to stand up quickly within 8 s and to remain in the upright position for another 3 min. The CBFV curve was dynamically plotted. The CBFV variation was calculated by subtracting the mean CBFV value in the upright position from the value in the supine position.

### Dynamic Cerebral Autoregulation Protocol

DCA measurement was performed as previously reported (17, 18). The participants were told to abstain from alcohol, nicotine, and caffeinated drinks for at least 12 h. The measurement was performed in a quiet, dedicated research laboratory at a controlled temperature of 22°C to 24°C. To minimize the diurnal variation of CA, all the participants were accessed at the same time of a day. First, the subjects were told to breathe normally in a supine position for 15 min to measure baseline arterial BP (Omron 711) and heart rates. Then, the continuous bilateral MCA blood flow velocity (MultiDop X2, DWL, Sipplingen, Germany) and continuous finger arterial BP (Finometer Model 1, FM, Netherlands) were recorded simultaneously for 10 min in a supine position. End-tidal CO_2_ was measured using a capnograph with a face mask attached to the nasal cannula. All the measurements were performed by one experienced operator.

### Data Analysis

The dCA analysis of the clinical data was performed blindly for each subject. The data analysis of dCA was performed as previously reported using transfer function analysis (17, 19). For each recording, arterial BP and bilateral cerebral artery blood flow velocity were divided into a number of data segments by a 60-s window and overlapped for 30 s. Transfer function analysis of one segment of arterial BP and bilateral cerebral artery blood flow velocity was carried out as,

(1) $$H\left(f\right)=\frac{S_{pv}\left(f\right)}{S_{pp}\left(f\right)},$$

where *H(f)* denotes the frequency response. *S_pp_(f)* is the auto-spectrum of arterial BP and *S_pv_(f)* is the cross-spectrum between arterial BP and cerebral artery blood flow velocity. For each subject, *S_pp_(f)* and *S_pv_(f)* were averaged over the segments to improve statistical reliability. The gain *|H(f)|* and phase difference ø*(f)* can then be computed as,

(2) $$|H(f)|=\sqrt{\left\{|H_{R}(f){|}^{2}+|H_{I}(f){|}^{2}\right\}},$$

(3) $$ø(f)=\tan^{-1}\left[\frac{H_{I}(f)}{H_{R}(f)}\right],$$

where *H_R_(f)* and *H_I_(f)* are the real and imaginary parts of *H(f)*, respectively. The phase difference and gain were analyzed in low-frequency band (0.07–0.2 Hz) (20). The data with coherence <0.4 were excluded due to the insufficient quality.

### Statistical Analysis

Data were analyzed using the Statistical Package for the Social Sciences version 23.0 (SPSS, IBM, West Grove, PA, USA). Continuous data were expressed as the mean and standard deviation, and the discrete variables were expressed as the rate (percentage). Normality of distribution for continuous variables was evaluated using the Kolmogorov–Smirnov goodness-of-fit test. Student’ *t*-tests and paired *t*-tests were used to evaluate the measurement data. Nonparametric Mann–Whitney *U* tests were performed for variables that were not distributed normally. Chi-squared and Fisher’s exact tests were used to examine the count data. Univariate and multivariate linear regression were used to assess the association of polysomnographic findings and dCA parameters within the IRBD group. The level of significance was set at *P* < 0.05.

## Results

### Demographic and Clinical Information

In total, 66 participants were enrolled in this study, including 30 IRBD patients and 36 controls. No significant differences were detected between the IRBD patients and controls as for smoking, drinking, hypertension, diabetes, and hyperlipemia. The demographic and clinical characteristics of all the participants are listed in **Table 1**. PSG parameters of the IRBD patients are presented in **Table 2**.

**Table 1:** Demographical characteristics, clinical features, and dCA parameters of IRBD patients and control group.

	IRBD patients (n = 30)	Control group (n = 36)	*P* value
Sex (male/female)	18/12	24/12	0.615
Age (years)	56.5 (45.75–63.25)	54 (47–62.75)	0.812
Age at onset (years)	48.5 (40.75–58)	—	—
Interval of symptoms to diagnosis (years)	3.5 (1–8.5)	—	—
Education (years)	10.5 (9–16)	12 (9–15)	0.789
Smoke (%)	11 (36.7)	14(38.9)	1.000
Drink (%)	9 (30.0)	11 (30.6)	1.000
Hypertension (%)	9 (30.0)	9 (25)	0.783
Diabetes (%)	5 (16.7)	2 (5.6)	0.231
Hyperlipemia (%)	5 (16.7)	3 (8.3)	0.452
MBP (mmHg)	91.86 ± 9.52	89.65 ± 8.32	0.323
Mean CBFV (cm/s)			
Right	65.80 ± 17.28	63.34 ± 11.61	0.436
Left	66.15 ± 15.79	64.99 ± 13.83	0.510
Variation of CBFV	7.40 ± 5.91	—	—
EtCO_2_ (mmHg)	36 (34.05–36.925)	36.1 (35.025–37.45)	0.297
HR (bpm)	71.20 ± 8.64	70.81 ± 9.48	0.862
Phase difference (degrees)	44.47 ± 18.84	55.72 ± 16.74	0.013*
Gain (cm/s/mmHg)	0.78 ± 0.29	0.70 ± 0.22	0.193

IRBD, idiopathic REM behavior disorder; MBP, mean blood pressure; CBFV, cerebral blood flow velocity; MCA, middle cerebral artery; EtCO_2_, end-tidal CO_2_; HR, heart rate. *P < 0.05 compared with control group.

**Table 2:** PSG parameters of IRBD patients.

Total sleep time (min)	411.52 ± 83.37 (226–568)
Sleep onset latency (min)	14.85 ± 18.79 (0–63.4)
REM latency (min)	130.30 ± 70.23 (27–282.5)
Sleep efficiency (%)	77.87 ± 9.95 (55–96.8)
Stage 1 NREM (%)	20.08 ± 8.62 (4.6–40.7)
Stage 2 NREM (%)	55.81 ± 11.38 (24.6–88.2)
Stage 3 NREM (%)	5.47 ± 4.22 (0–15)
REM sleep (%)	18.61 ± 7.06 (4.3–33.0)
PLMS index	29.08 ± 50.92 (0–208.7)
Tonic EMG activity (%)	10.86 ± 8.93 (2.08–27.71)
Phasic EMG activity (%)	15.82 ± 14.56 (0.5–45.83)
Arousal index	17.56 ± 9.28 (3.4–43.0)
AHI	3.57 ± 3.19 (0–9.8)

IRBD, idiopathic REM behavior disorder; REM, rapid eye movement; NREM, non-REM sleep; PLMS, periodic limb movement during sleep; EMG, electromyography; AHI, apnea-hypopnea index. Parentheses indicate a value range (min.–max.).

### Dynamic Cerebral Autoregulation in the IRBD Patients Versus Control Group

**Figure 1:**
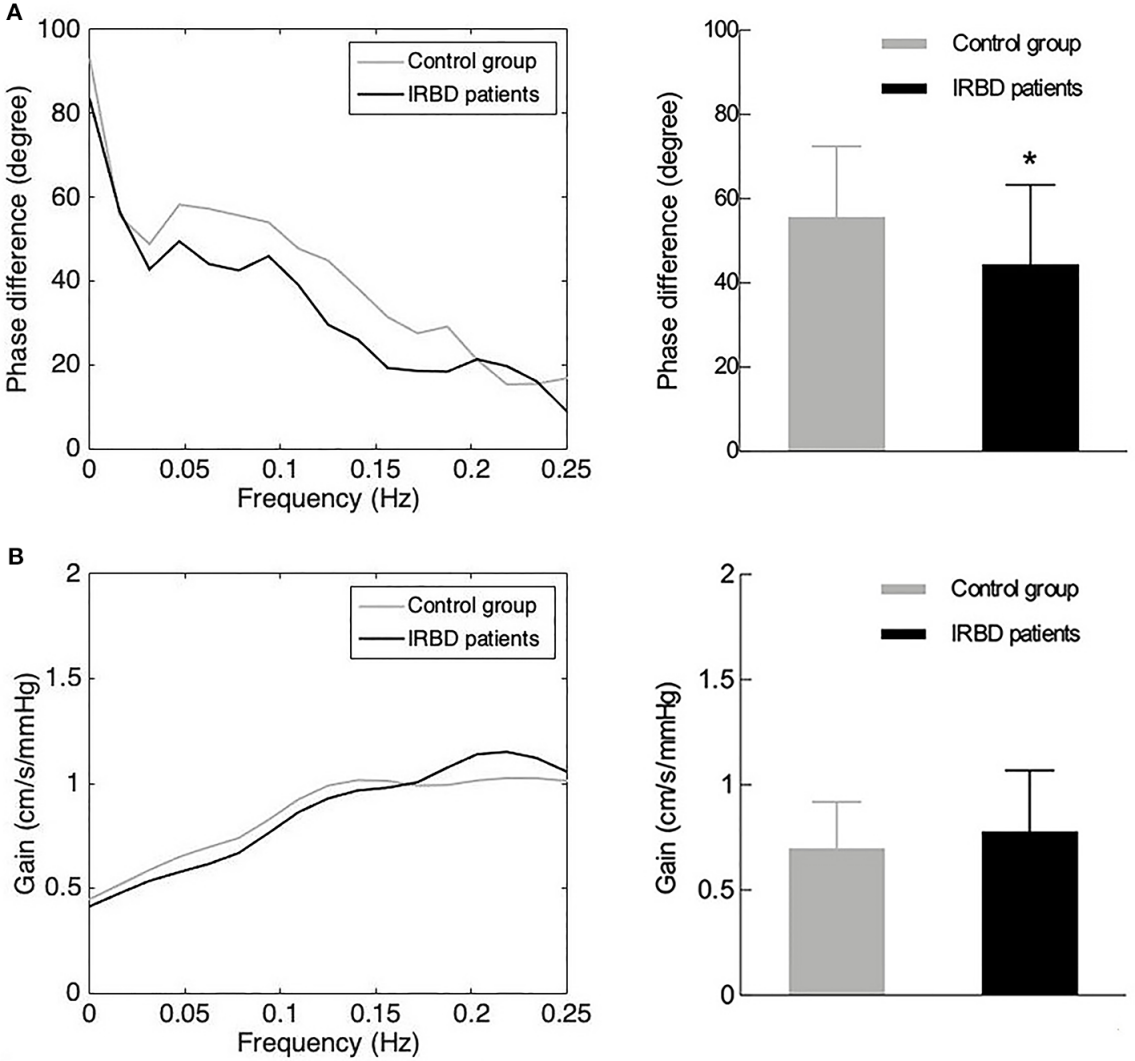
DCA parameters in IRBD patients and control group. **(A)** Phase difference in the frequency domain (left side) and its statistical distributions (right side) in IRBD patients and control group. **(B)** Gain in the frequency domain (left side) and its statistical distributions (right side) in IRBD patients and control group. Bars denote means, whiskers denote standard error. *Represents for statistically different (*P* < 0.05).

Comparison of hemodynamic and dCA parameters between the two groups are presented in **Table 1**. There were no significant differences between the right and left MCA dCA parameters (phase difference and gain) of both the groups. Therefore, averaged values were used in the subsequent analysis. IRBD patients had significantly lower phase difference (*P* = 0.013) than the control group. The gain value of the IRBD patients was higher than the control group, but the difference did not reach statistical level (**Figure 1A, B**).

### Correlation Between PSG Variables and dCA Parameters

The clinical and PSG variables used in the univariable and multivariable analyses are shown in **Table 3**. In the univariable model, PLM index was inversely correlated to phase difference. Wake after sleep onset, REM latency and stage 1 NREM were positively related to gain. Variables with *P* < 0.1 after the univariable analysis were included in the multivariable model. In a multivariable analysis, no variables were found to be related to dCA parameters.

**Table 3:** Univariable and multivariable linear analysis between dynamic cerebral autoregulation parameters and PSG variables in IRBD patients.

	Univariable analysis				Multivariable analysis			
	Phase difference		Gain		Phase difference		Gain	
	β	*P* value	β	*P* value	β	*P* value	β	*P* value
Gender	−3.88	0.416	−0.026	0.699	4.998	0.497	−0.280	0.246
Age	−0.16	0.391	−0.003	0.276	0.069	0.806	−0.005	0.641
Variation of CBFV	−0.020	0.959	0.033	0.932				
Total sleep time	−0.016	0.712	<0.001	0.963				
Sleep onset latency	0.256	0.174	−0.002	0.440				
REM latency	0.026	0.388	0.002	0.010 ^ab^			0.002	0.365
WASO	−0.066	0.250	0.002	0.008 ^ab^			0.001	0.792
Sleep efficiency	0.106	0.769	−0.009	0.111				
REM sleep	−0.162	0.750	−0.004	0.656				
Stage 1 NREM	−0.209	0.616	0.013	0.033 ^ab^			0.012	0.461
Stage 2 NREM	0.239	0.447	−0.005	0.311				
Stage 3 NREM	0.168	0.593	0.002	0.900				
Arousal index	0.072	0.843	0.072	0.852				
PLMS index	−0.139	0.041^ab^	0.001	0.166	−0.129	0.091		
Tonic EMG activity	−0.298	0.609	0.003	0.770				
Phasic EMG activity	0.317	0.364	−0.010	0.069 ^a^			−0.009	0.204
AHI	0.076	0.947	0.027	0.109				

IRBD, idiopathic REM behavior disorder; REM, rapid eye movement; WASO, wake after sleep onset; NREM, non-REM sleep; PLMS, periodic limb movement during sleep; EMG, electromyography; AHI, apnea-hypopnea index. ^a^Nominally significant values (*P* < 0.1) included in the multivariable model; ^b^*P* < 0.05 (statistically different).

## Discussion

The aim of this study was to investigate CA in patients with IRBD. We found that phase difference was significantly impaired in the patients with IRBD compared with the control group, indicating disrupted autoregulation function. Compromised CA might be an intermediate link between IRBD and neurological symptoms and a potential therapeutic target in improving neurological symptoms of these patients.

Several studies have been performed to evaluate cerebral perfusion in the patients with RBD using SPECT, and both increased and reduced perfusion have been reported. Mazza et al. found increased perfusion in the pons and putamen bilaterally and in the right hippocampus, as well as decreased perfusion in frontal cortices in the IRBD patients (2). Similarly, Vendette et al. observed increased CBF in the bilateral pons, putamen, and hippocampus and decreased CBF in the frontal cortex and in medial parietal areas (4). They also detected cerebral perfusion in the patients with IRBD mild cognitive impairment, and found relative hypoperfusion in frontal regions, and hyperperfusion in the right hippocampus and parahippocampal gyri (21). However, the study of Hanyu and colleagues demonstrated decreased regional CBF (rCBF) in the parieto-occipital lobe (precuneus), limbic lobe, and cerebellar hemispheres in the patients with IRBD, but found no brain areas with significantly increased rCBF. A subsequent follow-up study of their team showed decreased rCBF in bilateral parietotemporal and occipital areas at the first and second SPECT and decreased rCBF in the medial portions of the parieto-occipital lobe at the second SPECT (22). Although the results were contradictory in these studies, all of them indicated the changes in brain perfusion in patients with RBD. The potential mechanisms of altered cerebral perfusion in RBD patients have not been fully understood yet. In our study, we gave a reasonable explanation—the impairment of CA was involved in both hypoperfusion and hyperperfusion of brain. Simultaneously, the impairment of CA might be associated with cognitive impairment in patients with RBD.

RBD is not only frequent in patients with neurodegenerative diseases such as PD, LBD, and MSA, but also links with early signs of these disorders (23, 24). Previous studies have investigated CA in patients with neurodegenerative diseases, including PD, MSA, and dementia (7, 8, 25). Because of different methods and criteria used in these studies, contrasting conclusions have been reached. To the best of our knowledge, CA in IRBD has not been reported yet. Our study found that CA was impaired in patients with IRBD. Although participants recruited in this study showed no manifestations of neurodegenerative diseases, they may have subtle signs of early degeneration. Thus, we cannot rule out the possibility that early neurodegenerative process may also be involved. Further researches will be required to determine whether CA dysfunction in patients with IRBD is the result of the disorder itself or also an epiphenomenon of the underlying neurodegenerative process. Anyway, this finding indicates that there might be a common mechanism underlying IRBD and these diseases.

Though the potential mechanisms about the impact of IRBD on CA remain unclear, there is a theoretical possibility that the patients with IRBD have impaired CA. First, dysfunction of one or several neuronal pathways in the brainstem is involved in the patients with IRBD, including nigrostriatal dopaminergic neurons, noradrenergic or cholinergic neurons of the locus coeruleus or subcoeruleus complex, serotoninergic neurons of the raphe nucleus, and cholinergic neurons of the pedunculopontine nucleus. The variation in neurotransmitter concentrations [norepinephrine (26), dopamine (27), serotonin, acetylcholine (28), and gamma-aminobutyric acid (29), etc.] resulting from the dysfunction of these pathways are also involved in vasomotor function, which is a control mechanism of CA (**Supplemental Figure 1**). Second, autonomic dysfunction, which is frequently present in patients with IRBD (30), has an impact on CA. Autonomic function is compromised at the early phase of IRBD, so is autoregulation impairment. Sympathetic nervous system modulates CA by cerebral vasodilatation or vasoconstriction (31). The study of Postuma et al. demonstrated that the autonomic dysfunction in IRBD was identical in patients who would or would not develop defined neurodegenerative disease, suggesting that autonomic dysfunction is linked with IRBD dependently (32).

This study had several limitations. First, although carefully examined by neurologists, the IRBD patients and controls did not undergo the motor part of the Unified PD Rating Scale. Thus, very subtle parkinsonian signs might have been missed. Second, the correlation between dCA parameters and sign of early degeneration was not explored in this study. Third, this study did not include longitudinal data on follow-up conversion to neurodegenerative disease. The correlation between dCA parameters and development of neurodegenerative disease should be explored in future study. In addition, this was an observational study of a small sample size without in-depth mechanism research. Further studies with a larger sample size and animal studies are needed.

## Conclusion

Patients with IRBD exhibited impaired dCA, which might be an intermediate link between IRBD and neurological symptoms.

## Data Availability Statement

The datasets generated for this study are available on request to the corresponding authors.

## Ethics Statement

The studies involving human participants were reviewed and approved by the ethics committee of the First Hospital of Jilin University. The patients/participants provided their written informed consent to participate in this study.

## Author Contributions

Drafted the manuscript: SL, ZW. Revised the manuscript: SL, Z-NG. Drew the figures: JL, FD. Acquisition of data: HJ, XS, MJ, YL. Data analysis: SL, JL. Statistical analysis: SL, ZW. Conceived and designed the manuscript: Z-NG, YY. All authors read and approved the final manuscript.

## Funding

This article was supported by the National Natural Science Foundation of China to Yi Yang (Grant No. 81571123), the National Key R&D Program of China (2016YFC1301600), and JLUSTIRT (2017TD-12) to Yi Yang.

## Conflict of Interest

The authors declare that the research was conducted in the absence of any commercial or financial relationships that could be construed as a potential conflict of interest.

## Supplementary Material

Supplementary Figure 1 Dysfunction of several neuronal pathways in the brainstem that involved in the patients with IRBD. Dysfunction of nigrostriatal dopaminergic neurons, noradrenergic or cholinergic neurons of the locus coeruleus or subcoeruleus complex, serotoninergic neurons of the raphe nucleus, and cholinergic neurons of the pedunculopontine nucleus are involved in the patients with IRBD, resulting in the variation of several neurotransmitter concentrations, including norepinephrine, dopamine, serotonin, acetylcholine, and GABA. Alteration of these neurotransmitters, which are also involved in vasomotor function, result in impairment of CA.
