# Supplementary figures and images for: Compromised Dynamic Cerebral Autoregulation in Patients With Idiopathic Rapid Eye Movement Behavior Disorder: A Case-Control Study Using Transcranial Doppler
Source: Front Psychiatry. 2020 Feb 19;11:51. doi: 10.3389/fpsyt.2020.00051 (PMC7042385; doi:10.3389/fpsyt.2020.00051)

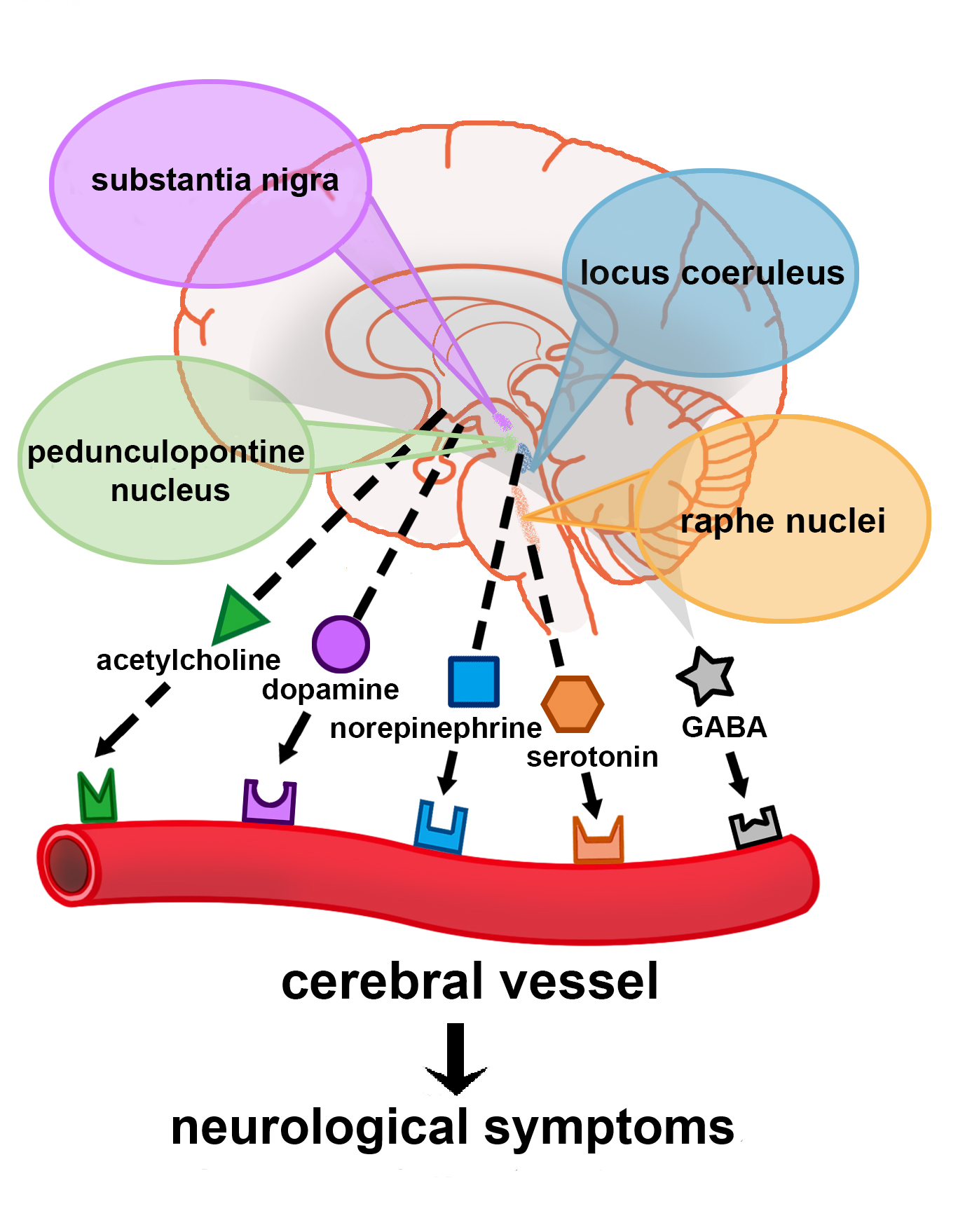

Supplement: Supplementary Figure 1 — Dysfunction of several neuronal pathways in the brainstem that involved in the patients with IRBD. Dysfunction of nigrostriatal dopaminergic neurons, noradrenergic or cholinergic neurons of the locus coeruleus or subcoeruleus complex, serotoninergic neurons of the raphe nucleus, and cholinergic neurons of the pedunculopontine nucleus are involved in the patients with IRBD, resulting in the variation of several neurotransmitter concentrations, including norepinephrine, dopamine, serotonin, acetylcholine, and GABA. Alteration of these neurotransmitters, which are also involved in vasomotor function, result in impairment of CA. [file Image_1.jpeg]
